# Comparisons of clinical characteristics, prognosis, epidemiological factors, and genetic susceptibility between HER2‐low and HER2‐zero breast cancer among Chinese females

**DOI:** 10.1002/cam4.6129

**Published:** 2023-06-30

**Authors:** Lu Zheng, Yunmeng Zhang, Zhipeng Wang, Huan Wang, Chunfang Hao, Chenyang Li, Yanrui Zhao, Zhangyan Lyu, Fangfang Song, Kexin Chen, Yubei Huang, Fengju Song

**Affiliations:** ^1^ Department of Epidemiology and Biostatistics, Tianjin's Key Laboratory of Molecular Cancer Epidemiology, National Clinical Research Center for Cancer Tianjin Medical University Cancer Institute and Hospital, Tianjin Medical University Tianjin China; ^2^ Department of Infectious Disease Control and Prevention Heping Centers for Disease Control and Prevention of Tianjin Tianjin China; ^3^ Department of Breast Cancer, National Clinical Research Center for Cancer Tianjin Medical University Cancer Institute and Hospital, Tianjin Medical University Tianjin China

**Keywords:** breast cancer, HER2‐low BC, HER2‐zero BC, polygenic risk score, prognosis, SNPs

## Abstract

**Background:**

Traditional human epidermal growth factor receptor 2 (HER2)‐negative breast cancer (BC) is recommended to be divided into HER2‐low and HER2‐zero subtypes due to different prognosis. However, few studies investigated their differences in clinical characteristics and prognosis among Chinese HER2‐negative BC and their stratified differences by hormone receptor (HR), while fewer studies investigated their differences in epidemiological factors and genetic susceptibility.

**Methods:**

A total of 11,911 HER2‐negative BC were included to compare the clinical characteristics and prognosis between HER2‐zero and HER2‐low BC, and 4227 of the 11,911 HER2‐negative BC were further compared to 5653 controls to investigate subtype‐specific epidemiological factors and single nucleotide polymorphisms(SNPs).

**Results:**

Overall, 64.2% of HER2‐negative BC were HER2‐low BC, and the stratified proportions of HER2‐low BC were 61.9% and 75.2% for HR‐positive and HR‐negative BC, respectively. Compared to HER2‐zero BC, HER2‐low BC among HR‐positive BC showed younger age at diagnosis, later stage, poorer differentiation, and higher Ki‐67, while elder age at diagnosis and lower mortality were observed for HER2‐low BC among HR‐negative BC (all *p* values <0.05). Compared to healthy controls, both HER2‐low and HER2‐zero BC are associated with similar epidemiological factors and SNPs. However, stronger interaction between epidemiological factors and polygenic risk scores were observed for HER2‐zero BC than HER2‐low BC among either HR‐positive [odds ratios: 10.71 (7.55–15.17) and 8.84 (6.19–12.62) for the highest risk group compared to the lowest risk group] or HR‐negative BC [7.00 (3.14–15.63) and 5.70 (3.26–9.98)].

**Conclusions:**

HER2‐low BC should deserve more attention than HER2‐zero BC, especially in HR‐negative BC, due to larger proportion, less clinical heterogeneity, better prognosis, and less susceptibility to risk factors.

## 1 BACKGROUND

Breast cancer (BC) has surpassed lung cancer as the most common cancer in most countries and the leading cause of cancer deaths in several countries in recent years, including China.^1^, ^2^, ^3^ An estimated of 2.3 million BC cases and 685,000 BC deaths occurred in 2020.^1^ Although the current BC incidence rate is lower than the global average incidence rate, BC in China accounted for 18.4% of newly diagnosed BC cases and 17.1% of BC deaths across the whole world in 2020.^1^ Meanwhile, China would face a potentially BC burden due to the decreasing age at menarche, decreasing number of parity, decreasing months of breastfeeding, increasing age at first birth, increasing birth interval, and increasing age at menopause.^4^ Despite there was an obvious improvement in overall 5‐year survival rate of BC in China during the past decade,^5^, ^6^ there was no obvious increase in early‐stage BC among clinic‐detected BC due to delayed treatment and lack of regular screening.^7^, ^8^ Decreasing the burden of BC remains a challenge faced by many countries, and identification of BC subtypes with better prognosis and implementation of comprehensive interventions based on modifiable risk factors would be potentially suitable strategies.

Human epidermal growth factor receptor 2 (HER2) is a prototype oncogene and is overexpressed in 10%–30% of invasive BC.^9^, ^10^, ^11^ However, approximately 50%–60% of HER2‐negative metastatic BC expressed low levels of HER2 [immunohistochemical (IHC) 1+/2+ and negative results on in situ hybridization (ISH)]^12^, ^13^ and were defined as HER2‐low BC. Recently, HER2‐low BC has been suggested as a potentially independent subtype of BC from traditionally HER2‐negative BC [including HER2‐low BC and HER2‐zero BC (IHC of 0)] due to a potential better prognosis.^9^, ^14^ Before the HER2‐directed antibody‐drug conjugates (ADC) with chemotherapeutics were developed, HER2‐low BC is usually treated as HER2‐negative BC and does not have the option to receive targeted treatment.^13^, ^15^ However, emerging shreds of evidence suggested that HER2‐low BC can benefit from ADC treatment. The latest DESTINY‐Breast04 trial showed that trastuzumab deruxtecan (T‐DXd) resulted in significantly longer progression‐free and overall survival than the physician's choice of chemotherapy.^16^ Trastuzumab duocarmazine (SYD985) has also shown very promising therapeutic activity in HER2‐low BC.^17^, ^18^ Therefore, identification of HER2‐low BC and investigating the potential risk factors associated with HER2‐low BC would be very important to improve the survival and promote the prevention of BC. However, few studies investigated the differences in clinical characteristics and prognosis between HER2‐low and HER2‐zero BC among Chinese HER2‐negative BC and their stratified differences by hormone receptor (HR), while fewer studies investigated their differences in epidemiological factors and genetic susceptibility.

Therefore, in this study, we first aimed to compare the clinical characteristics and prognosis between HER2‐zero and HER2‐low BC based on the prospective Tianjin Breast Cancer Cases Cohort (TBCCC). To further investigate subtype‐specific epidemiological factors and genetic susceptibility, we conducted subtype‐specific case–control studies based on patients with HER2‐zero and HER2‐low BC from above TBCCC and healthy controls from the Multi‐modality Independent Screening Trial (MIST) for breast cancer.

## 2 METHODS

### 2.1 Study sources and population selection

Detailed information on TBCCC has been described in previous studies.^19^, ^20^, ^21^, ^22^ Briefly, TBCCC was an open cohort that aimed to investigate the long‐term survival of Chinese patients with female BC and tried to identify novel markers associated with BC prognosis. All newly diagnosed and pathologically confirmed patients with BC in Tianjin Medical University Cancer Institute and Hospital (TMUCIH) were invited since January 2007. After admission to TMUCIH and informed consent, demographic and epidemiologic information was collected by a full‐time physician with a face‐to‐face interview based on a structured questionnaire. A total of 5–10 milliliters of blood samples were suggested to collect for all patients after questionnaire interviews. Information of date at diagnosis, cancer stage, grade, histopathologic type, common IHC markers [including estrogen receptor (ER), progesterone receptor (PR), HER‐2, Ki‐67, cytokeratin 5/6, epidermal growth factor receptor], and primary treatments (endocrine therapy, chemotherapy, and radiotherapy) were collected and recorded on uniform case report form within 1 week after discharge. Positive HR was defined as positive ER or PR, otherwise, it was defined as negative HR. Due to limited treatment options for traditional HER2‐negative BC or even HER2‐low BC, the voluntary principle ISH testing, and the high cost and inaccessibility of novel ADC therapy, many patients choose not to undergo ISH testing after IHC test. Therefore, there was a large proportion of missing data in ISH testing. In this context, HER2‐low was simply defined as IHC 1+/2+, while HER2‐zero was defined as IHC of 0. Deaths were primarily ascertained by annual telephone follow‐up and supplemented by periodic linkage to the local cancer registry and death registry up to December 2021. The prognosis analyses were censored at the dates of death, lost to follow‐up, or end of the follow‐up period, whichever came first. In this study, further exclusion criteria included: (1) previous patients with BC before recruitment; (2) male patients with BC; (3) patients with contralateral breast cancer; (4) patients with HER2 overexpression (IHC 3+); (5) patients lost to follow‐up; (6) patients without enough blood sample to test biomarkers (only for the case–control study).

Detailed information on MIST has also been described in other previous studies.^7^, ^23^, ^24^, ^25^ Briefly, MIST was a multicenter BC screening trial that aimed to compare the performances of three BC screening modalities among Chinese females and further investigate the long‐term benefits of BC mortality. Asymptomatic women aged 45–65 years and living in local communities for at least 3 years in five cities (Tianjin, Beijing, Liaoning, Nanchang, and Feicheng) were invited to MIST between July 2008 and December 2010. After informed consent and a face‐to‐face questionnaire interview to collect demographic and epidemiologic information, all participants were invited to receive a clinical breast examination, breast ultrasound, and mammography within the same day. Physicians performed and interpreted the three examinations independently and blindly. All examinations followed unified technical protocols developed by the expert committees of MIST. Any positive screens with suspicious malignancy and highly suggestive of BC from the above three modalities were immediately recommended for pathological examination. BC cases were primarily ascertained by routine follow‐up after positive screens and supplemented by linkage to the local cancer registry up to September 2015. A total of 5–10 milliliters of blood samples were further collected from participants in Tianjin and Feicheng, and these participants were further followed up until December 2021. In this study, healthy women without a diagnosis of BC from Tianjin were matched to patients with BC from TBCCC based on the same region, the period between 2007 and 2015, and baseline age at the entrance with a difference of ±5 years at an approximate 1:1 ratio. Further exclusion criteria included: (1) any patients with BC diagnosed by pathologists, physicians, cancer registry, death registry, or self‐report during follow‐up; (2) previous patients with BC before recruitment; (3) participants without enough blood samples; (4) participants with suspicious BC but no enough information to exclude the diagnosis of BC.

Based on the above inclusion criteria, a total of 11,911 HER2‐negative BC from the TBCCC were selected for the cohort study, while 4227 of the 11,911 HER2‐negative BC and 5653 healthy controls from MIST were eligible for case–control study. Both TBCCC and MIST were approved by the Institutional Review Board of TMUCIH.

### 2.2 Demographic characteristics and epidemiological information

In both TBCCC and MIST, similar baseline questionnaires were developed and used to collect information on demographics (age, sex, race, marital status, education, income, insurance, etc.), family history of cancer, history of benign breast disease, hormonal and reproductive factors [age at menarche, menopausal status, age at menopause, history of abortion, oral contraceptives (OC), hormone replacement therapy (HRT), etc.], diet and lifestyle (tea consumption, alcohol consumption, smoking, physical activity, etc.), and social/psychological characteristics for both cases and controls. Ever alcohol consumption was defined as at least 50 mL of liquor per week. Ever smoking was defined as at least one cigarette per day for at least 3 months. Body weight (kg) and height (m) were measured by trained investigators, and body mass index (BMI) was calculated as weight in kilograms divided by the square of height in meters (kg/m^2^). All missing data in the above index variables were recoded as an independent group.

### 2.3 Selection of SNPs and genotyping

Up to October 2020, after systematically searching in PubMed, Embase (Ovid), and GWAS Integrator, a total of 25 single nucleotide polymorphisms (SNPs) identified from genome‐wide association studies (GWAS) were found to be specifically associated with Chinese female BC, including 9 SNPs originally identified from Chinese or East Asian ancestors,^26^, ^27^, ^28^, ^29^, ^30^ and 16 SNPs from European ancestors but further validated in large Chinese or East Asian population.^31^, ^32^, ^33^, ^34^, ^35^, ^36^ After excluding 2 SNPs with minor allele frequency (MAF) <0.05 and high linkage disequilibrium with other SNPs (*r* ^2^ > 0.8) and one SNP (rs6472903) that was not successfully genotyped in most samples, a total of 22 SNPs were finally successfully genotyped and included in this study, including rs616488, rs1219648, rs1292011, rs1432679, rs2046210, rs2236007, rs2290203, rs4784227, rs4849887, rs4951011, rs4973768, rs7107217, rs7697210, rs9485372, rs9693444, rs10474352, rs10771399, rs10822013, rs10941679, rs16857609, rs17356907, and rs17817449. Details of these SNPs are available in Table S1.

Leukocytes were separated from the collected plasma and stored in a cryotube at −80°C for DNA extraction. The QIAGEN DNA extraction kit (QIAGEN Inc.) was used to extract genomic DNA,^20^, ^37^ and the Wafergen SmartChip platform was used to genotype the targeted 22 SNPs. To ensure the accuracy and reliability of the genotyping results, approximately 5% of the samples were randomly selected for retesting.

### 2.4 Statistical analysis

Chi‐square tests were used to compare the differences in important clinical characteristics [including age, HR (only for analyses on overall BC), pTNM, grade, and Ki‐67 expression in IHC] and primary treatments (including endocrine therapy, chemotherapy, and radiotherapy) between HER2‐zero and HER2‐low BC, and further analyses were conducted to compare baseline demographic characteristics, epidemiological risk factors, and SNPs between BC cases and controls.

For the prognosis analyses, Kaplan–Meier curves were first used to estimate the cumulative mortality of HER2‐low and HER2‐zero BCs, and logrank tests were used to compare the overall mortality between the two subtypes of BCs. Multivariate Cox proportional hazard regression models were further used to compare the prognosis of the two subtypes of BCs after adjusting the all available clinical characteristics and primary treatments. The relative risks were measured as hazard ratios and 95% confidence intervals. Stratified comparisons were further conducted by HR status.

Based on the case–control study, due to the previous weak associations between each genetic SNP and overall BC, the univariate logistic regression model was first used to validate the preliminary associations between each SNP (exposure defined as heterozygote or rare homozygotes, and non‐exposure as wild‐type) and subtype‐specific BC. The polygenic risk scores (PRS) were calculated as the sum of risk alleles from all index SNPs.^38^, ^39^ Multivariable logistic regression models were used to evaluate the independent associations of traditional risk factors with overall and subtype‐specific BC. The associations were measured with odds ratios and 95% confidence intervals [ORs (95%CI)], and the nomograms were used to calculate the established risk factor scores (ERS). Kruskal–Wallis tests were used to determine whether there was a statistically significant difference in the medians of ERS and PRS between HER2‐low BC, HER2‐zero BC, and controls. Finally, participants were further divided into 16 subgroups according to the quartiles of both ERS and PRS, and further logistic regression models were conducted to test whether there was an interaction between ERS and PRS with subtype‐specific BC. Stratified analyses were also conducted according to HR status. To ensure comparability across different subgroups, uniform and subtype‐specific ERS and PRS were developed and used for subtype‐specific analyses.

All tests were two‐sided and *p* < 0.05 was considered statistically significant. All statistical analyses were conducted by R v.4.1.2 software (R Project for Statistical Computing) and SPSS v.26.0 (IBM Corporation).

## 3 RESULTS

### 3.1 Comparisons of clinical characteristics between HER2‐low and HER2‐zero BC

Overall, among 11,911 BCs with HER2‐negative BC, 64.2% (7646 patients) were HER2‐low BC, while the remaining 35.8% (4265 patients) were HER2‐zero BC. After stratifying by HR status, the proportions of HER2‐low BC were 61.9% (6135/9902) and 75.2% (1511/2009) for HR‐positive BC and HR‐negative BC, respectively.

As presented in Table 1, overall, compared to HER2‐zero BC, HER2‐low BC cases seemed to have a younger age at diagnosis (>60 years old, 20.5% vs. 22.0%, *p* value <0.001), later cancer stage (III‐IV, 20.0% vs. 18.4%, *p* value = 0.011), poorer differentiation (grade 3, 18.1% vs. 13.5%, *p* value <0.001), higher expression of Ki‐67 (>14%, 81.9% vs. 67.4%, *p* value <0.001), and received less endocrine therapy (28.1% vs. 33.7%, *p* value <0.001), more chemotherapy (88.3% vs. 83.6%, *p* value <0.001), and more radiotherapy (27.9% vs. 22.6%, *p* value <0.001). After stratifying by HR status, the differences in clinical characteristics between HER2‐low and HER2‐zero BC were more pronounced among HR‐positive BC, while only elder age at diagnosis was observed in HER2‐low BC compared to HER2‐zero BC among HR‐negative BC (Table 1).

**TABLE 1:** Comparisons of clinical characteristics between HER2‐zero and HER2‐low breast cancer (BC) by HR status.

Characteristics^a^	Group	Overall BC (*N* = 11,911)		*p* value^b^	HR+ BC (*N* = 9902)		*p* value^b^	HR‐ BC (*N* = 2009)		*p* value^b^
		HER2‐zero (*N* = 4265)	HER2‐low (*N* = 7646)		HER2‐zero (*N* = 3767)	HER2‐low (*N* = 6135)		HER2‐zero (*N* = 498)	HER2‐low (*N* = 1511)	
Age, years	≤40	586 (13.7)	1045 (13.7)	**<0.001**	503 (13.4)	875 (14.3)	**<0.001**	83 (16.7)	170 (11.3)	**<0.001**
	41–50	1505 (35.3)	2511 (32.9)		1345 (35.7)	2089 (34.1)		160 (32.1)	422 (28.0)	
	51–60	1234 (29.0)	2516 (33.0)		1058 (28.1)	1925 (31.4)		176 (35.3)	591 (39.2)	
	>60	937 (22.0)	1563 (20.5)		858 (22.8)	1237 (20.2)		79 (15.9)	326 (21.6)	
HR	HR−	498 (11.7)	1511 (19.8)	**<0.001**	—	—		—	—	
	HR+	3767 (88.3)	6135 (80.2)		—	—		—	—	
pTNM	0–I	1019 (35.7)	1943 (32.6)	**0.011**	912 (36.1)	1560 (32.5)	**0.007**	107 (32.6)	383 (33.2)	0.148
	II	1308 (45.8)	2823 (47.4)		1138 (45.1)	2282 (47.5)		170 (51.8)	541 (46.9)	
	III–IV	526 (18.4)	1194 (20.0)		475 (18.8)	965 (20.1)		51 (15.5)	229 (19.9)	
Grade	G1/G2	1548 (86.5)	4458 (81.9)	**<0.001**	1372 (94.2)	3869 (88.8)	**<0.001**	176 (52.9)	589 (54.3)	0.646
	G3	241 (13.5)	986 (18.1)		84 (5.8)	490 (11.2)		157 (47.1)	496 (45.7)	
Ki‐67	≤14%	1389 (32.6)	1387 (18.1)	**<0.001**	1349 (35.8)	1296 (21.1)	**<0.001**	40 (8.0)	91 (6.0)	0.115
	>14%	2876 (67.4)	6259 (81.9)		2418 (64.2)	4839 (78.9)		458 (92.0)	1420 (94.0)	
Endocrinotherapy	No	2623 (66.3)	4833 (71.9)	**<0.001**	2194 (62.2)	3607 (66.0)	**<0.001**	429 (99.1)	1226 (97.8)	0.100
	Yes	1336 (33.7)	1888 (28.1)		1332 (37.8)	1861 (34.0)		4 (0.9)	27 (2.2)	
Chemotherapy	No	691 (16.4)	877 (11.7)	**<0.001**	638 (17.2)	727 (12.1)	**<0.001**	53 (10.8)	150 (10.1)	0.690
	Yes	3515 (83.6)	6616 (88.3)		3076 (82.8)	5287 (87.9)		439 (89.2)	1329 (89.9)	
Radiotherapy	No	3123 (77.4)	5071 (72.1)	**<0.001**	2795 (78.2)	4081 (72.1)	**<0.001**	328 (71.1)	990 (72.4)	0.599
	Yes	912 (22.6)	1958 (27.9)		779 (21.8)	1581 (27.9)		133 (28.9)	377 (27.6)	

*Note*: G1/G2/G3, well/medium/poor differentiation.

^a^ Missing values in the index variable were not shown.

^b^ *p* value was calculated excludes missing values.

The bold values represent *p* <0.05.

### 3.2 Comparisons of prognosis between HER2‐low and HER2‐zero BC

**FIGURE 1:**
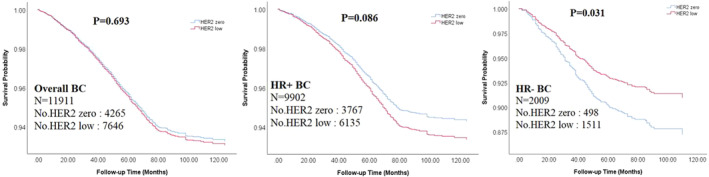
Kaplan–Meier survival curves for HER2‐zero and HER2‐low breast cancer (BC) by HR status. *p* values were calculated with multivariable COX regression after adjusting available clinical characteristics and primary treatments.

After a median follow‐up of 83 months, a total of 444 and 289 deaths were documented in HER2‐low and HER2‐zero BC, with crude mortality rates of 11.68 and 9.49 per 1000 person‐years, respectively (Table S2). Overall, the crude mortality of HER2‐low BC seemed to be significantly higher than HER2‐zero BC (*p* value = 0.013) (Table S2). After stratifying by HR status, a similar difference in mortality between HER2‐zero and HER2‐low BC was observed among HR‐positive BC (*p* value =0.010) but not among HR‐negative BC (*p* value =0.222) (Table S2). However, after adjusting available clinical characteristics (age at diagnosis, stage, grade, and Ki‐67) and primary treatments (endocrine therapy, chemotherapy, and radiotherapy), HER2‐low BC showed significantly lower mortality than HER2‐zero BC among HR‐negative BC, with a hazard ratio of 0.69 (95%CI: 0.50–0.97, *p* = 0.031) (Figure 1, Table S3).

### 3.3 Subtype‐specific associations of epidemiological factors with HER2‐low and HER2‐zero BC

As shown in Table S4, overall, compared to the healthy controls, both HER2‐low and HER2‐zero BC seemed to have higher BMI, more smokers, more BBD history, more family history of BC, more premenopausal status, more abortion, more OC, and more HRT. After stratifying by HR status, similar but more subtle differences difference were observed in HR‐negative BC than in HR‐positive BC (Table S4).

**FIGURE 2:**
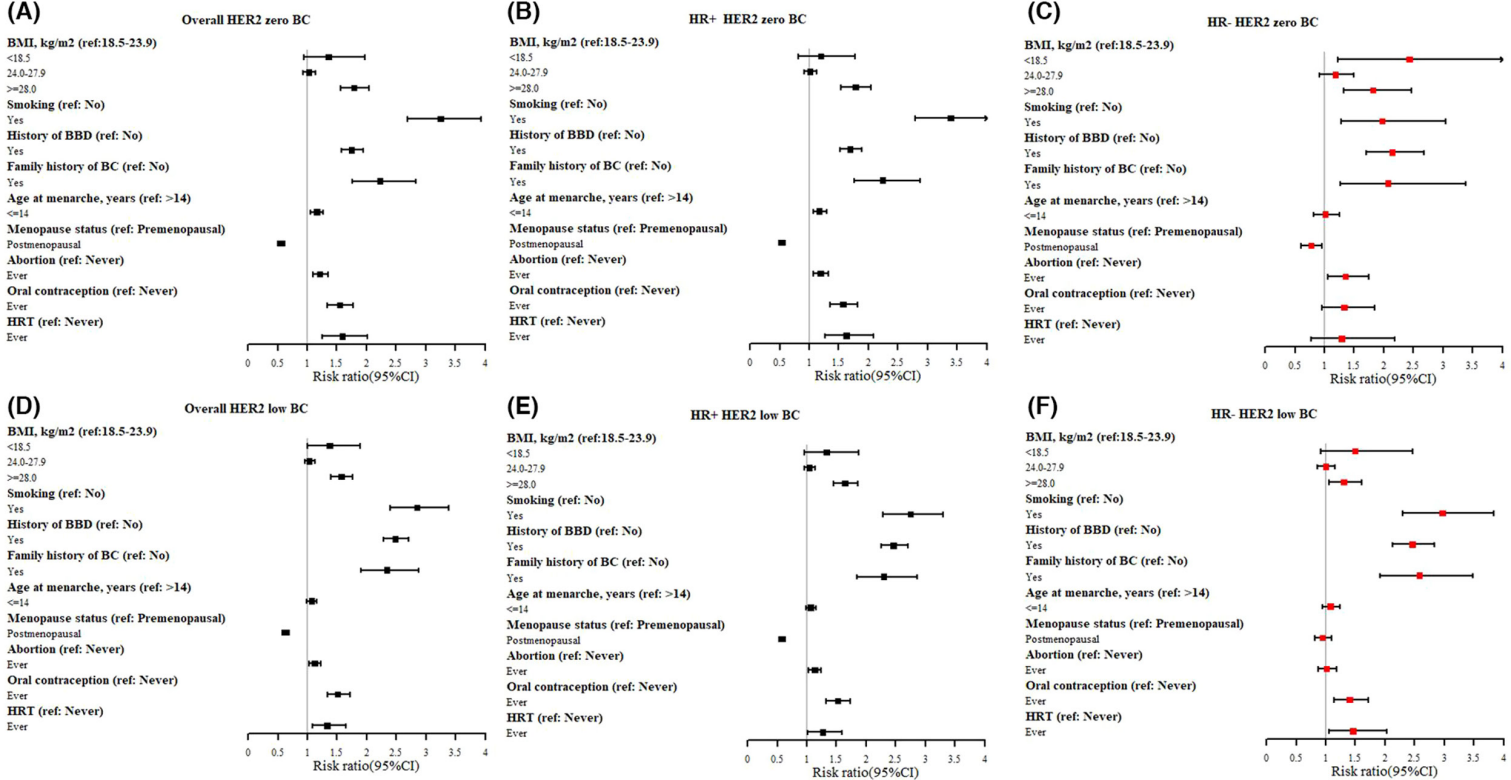
Adjusted relative risks of HER2‐zero and HER2‐low breast cancer (BC) by HR status with epidemiological factors. (A) Overall HER2 zero BC; (B) HR+ HER2 zero BC; (C) HR‐ HER2 zero BC; (D) Overall HER2 low BC; (E) HR+ HER2 low BC; (F) HR‐ HER2 low BC. *, missing values in the index variable were not shown.

As shown in Figure 2 and Table S5, compared to controls, based on the multivariable logistic regression, most epidemiological factors were similarly associated with HER2‐low and HER2‐zero BC among both HR‐positive and HR‐negative BC. However, some epidemiological factors differently associated with HER2‐low and HER2‐zero BC among HR‐negative BC. For example, low BMI (<18.5 kg/m^2^, OR [95% CIs]: 2.43 [1.22–4.87]), postmenopausal status (0.77 [0.61–0.96]) and history of abortion (1.35 [1.05–1.75]) were independently associated with HER2‐zero BC, while OC (1.40 [1.14–1.72]) and HRT (1.46 [1.06–2.03]) were independently associated with HER2‐low BC (Figure 2C and Table S5).

### 3.4 Subtype‐specific associations of genetic susceptibility with HER2‐low and HER2‐zero BC

**FIGURE 3:**
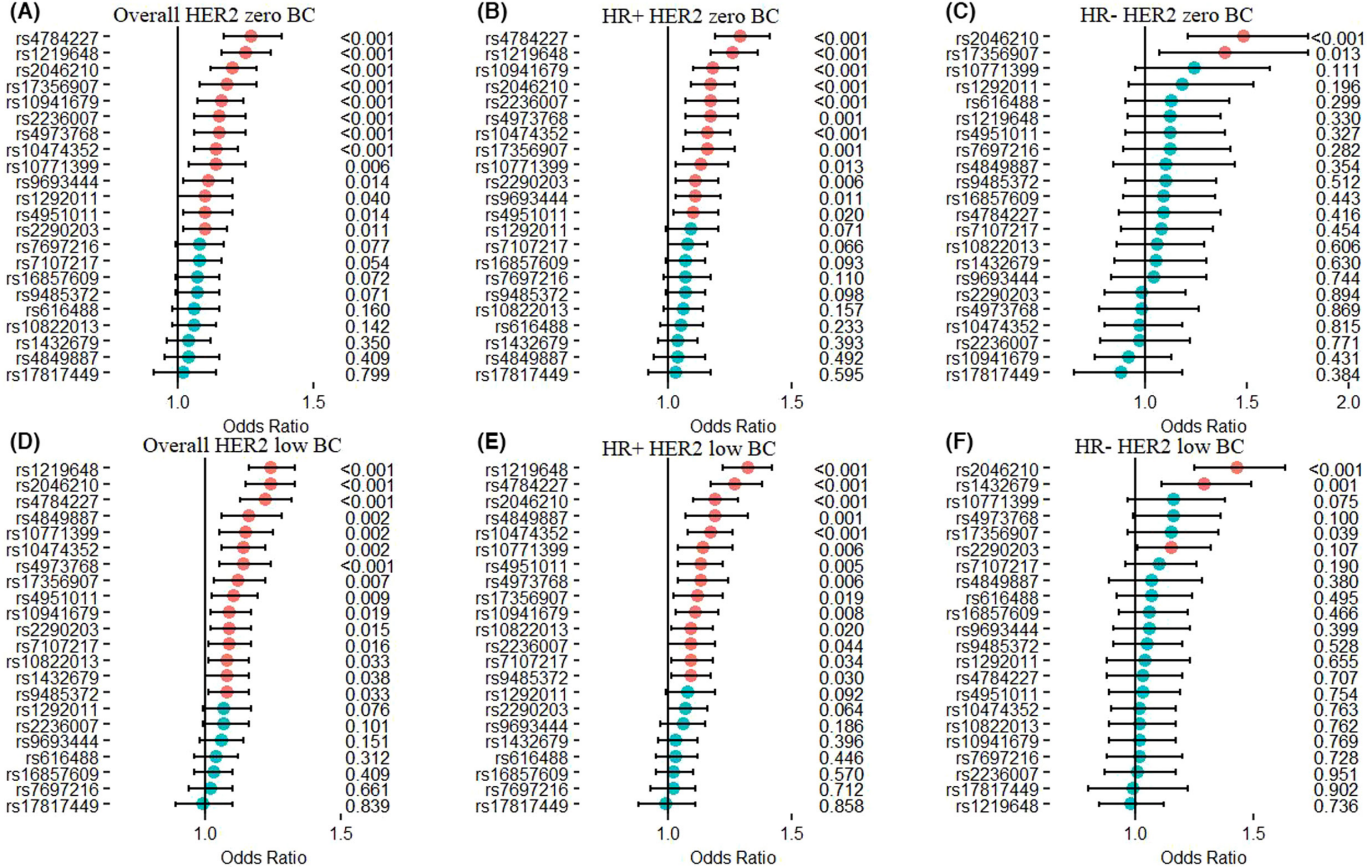
Unadjusted relative risks of HER2‐zero and HER2‐low breast cancer (BC) by HR status with GWAS‐identified SNPs.

Overall, compared to the controls, 13 of 22 selected SNPs were initially associated with the risk of HER2‐zero BC (ORs for heterozygote/rare homozygotes ranging from 1.10 to 1.27), while 15 SNPs were initially associated with HER2‐low BC (ORs ranging from 1.08 to 1.24) (Figure 3A,D; and Table S6). After stratifying by HR status, among HR‐positive BC, 12 and 14 SNPs significantly associated with HER2‐zero and HER2‐low BC, respectively. Among HR‐negative BC, only 2 (rs2046210 and rs17356907) and 3 SNPs (rs2046210, rs2290203, and rs1432679) are significantly associated with HER2‐zero and HER2‐low BC, respectively (Figure 3B,C,E,F; and Table S6).

### 3.5 Interaction of ERS and PRS on the risks of HER2‐zero and HER2‐low BC

**FIGURE 4:**
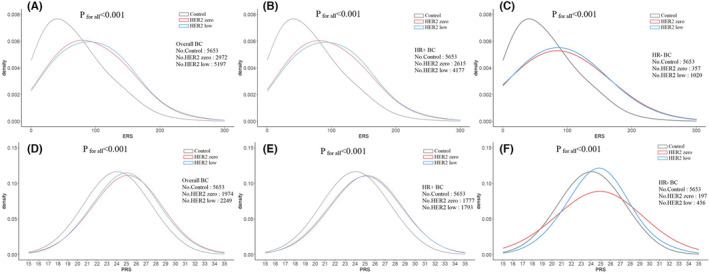
Distribution of ERS (A–C) and PRS (D–F) between HER2‐zero breast cancer (BC), HER2‐low BC, and healthy controls.

**FIGURE 5:**
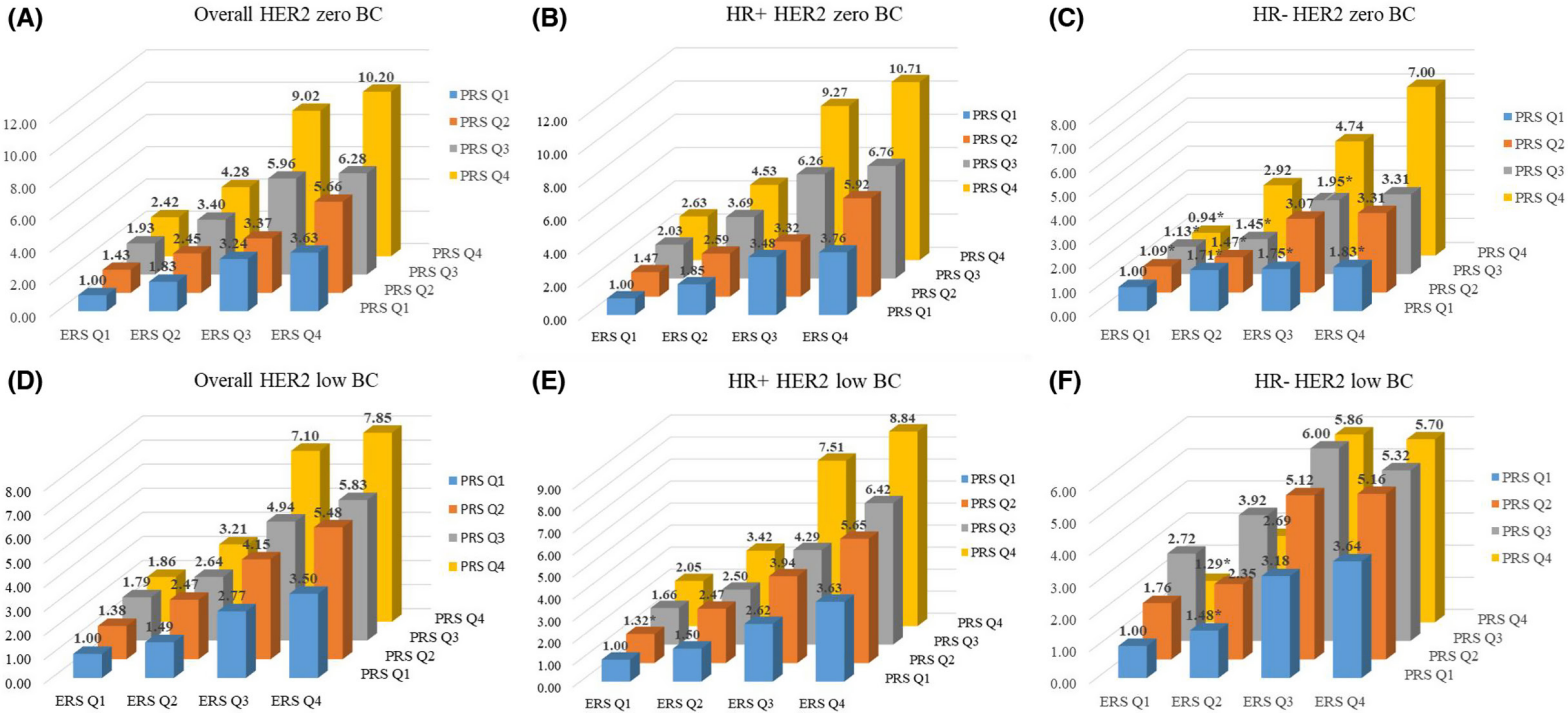
Interaction of ERS and PRS on the risks of HER2‐zero (A/B/C) and HER2‐low (D/E/F) breast cancer by HR status. *p > 0.05.

As shown in Figure 4 and Table S7 and S8, overall, both HER2‐low BC and HER2‐zero BC showed significantly higher ERS and PRS than healthy controls (both *p* values <0.001), while there was no obvious difference between HER2‐low and HER2‐zero BC. After stratifying by HR status, similar medians of ERS and PRS were still observed in both HER2‐low and HER2‐zero BC among HR‐positive BC and higher than those in healthy controls. However, among HR‐negative BC, obviously different kurtosis of ERS and PRS were observed between HER2‐zero and HER2‐low BC (Figure 4). Furthermore, an obvious interaction between ERS and PRS was observed on the risk of both HER2‐zero and HER2‐low BC. However, their interaction seemed to associate with higher risk of HER2‐zero BC (10.20 [7.29–14.28]) than HER2‐low BC (7.84 [5.60–10.99]) for the highest risk group. After stratifying by HR‐status, stronger interaction was still observed for HER2‐zero BC than HER2‐low BC among either HR‐positive ([7.55–15.17] and 8.84 [6.19–12.62] for the highest risk groups compared to the lowest risk group) or HR‐negative BC (7.00 [3.14–15.63] and 5.70 [3.26–9.98]) (Figure 5 and Table S9).

## 4 DISCUSSION

To the best of our knowledge, this was the first study to investigate the differences in epidemiological factors and genetic susceptibility between HER2‐low and HER2‐zero BC, especially for Chinese HER2‐negative BC. Although previous studies have investigated and compared the differences in clinical characteristics and prognosis between HER2‐low and HER2‐zero BC, the larger sample size and relatively longer follow‐up time corroborated this study with more credible results than previous studies. The subtype‐specific risk factors and genetic susceptibility would provide several preliminary insights into the established prevention and therapeutic strategy and trigger more investigations of HER2‐low and HER2‐zero BC in the future, especially for HR‐negative BC.^40^

Consistent with previous studies, more than half of BCs were qualified as HER2‐low BC in this study.^11^, ^14^, ^41^, ^42^ However, insistent with previous studies,^12^, ^40^, ^42^, ^43^, ^44^, ^45^ we observed a relatively high proportion of HER2‐low BC in traditionally HER2‐negative BC, especially in HR‐negative BC. Several reasons would potentially lead to the high proportion of HER2‐low in this study, including lack of ISH assay to reclassify BC with IHC 2+ as true HER2‐low BC or HER2‐positive BC, heterogeneity due to changes in testing HER2 protein expression with IHC between 2007 and 2021, and potential heterogeneity among different populations. Moreover, since TMUCIH was one of the best cancer centers in northern China, the bias of patients' self‐selection would also lead to more HER2‐low BC admitted to TMUCIH in the hope of more opportunities for potential targeted therapies specifically associated with HER2‐low BC. Therefore, how to improve the percentage of ISH assay among traditionally HER2‐negative BC and how to identify the HER2‐low BC from the traditionally HER2‐negative BC with more easy‐to‐use methods would be a big challenge faced by several countries.

Although anti‐HER2 therapy has not been regularly provided for patients with HER2‐low BC until now in China, we still observed a potentially better prognosis for HER2‐low BC than HER2‐zero BC, especially in HR‐negative BC, which was also observed in previous studies.^9^, ^42^, ^46^ The potentially better prognosis of HER2‐low/HR‐negative BC is likely to benefit from the fact that low HER2 expression provides potential non‐specific therapeutic response for routine treatments besides HER2‐targeted therapy. For HER2‐low/HR‐positive BC, although low HER2 expression could still provide potential non‐specific therapeutic response, the response would probably be weaker than the HR‐specific therapeutic response. Moreover, among HR‐positive BC, HER2‐low BC seems to show younger age at diagnosis, later stage, poorer differentiation, and higher Ki‐67 compared to HER2‐zero BC. Additionally, the crosstalk between HR signaling and HER2 signaling would also lead to therapy resistance to HER2‐low non‐specific therapeutic response.^47^ All of these factors would work together to dilute the non‐specific therapeutic response associated with low HER2 expression, and then lead to non‐differential prognosis between HER2‐low/HR‐positive and HER2‐zero/HR‐positive BC after adjusting for potential confounding factors. However, several studies also revealed similar prognosis between HER2‐low and zero BC regardless of HR status.^12^, ^42^, ^43^, ^48^ The heterogeneity may also be caused by the unstable expression of HER2 during BC progression, including the inconsistency between the primary tumor and matched advanced‐stage biopsy,^49^ and the discrepancy in HER2 status between primary tumors and matched relapse samples.^50^

Compared to the healthy controls, although there were no obvious differences in risk factors between HER2‐low and HER2‐zero BC among HR‐positive BC, there were subtle differences in subtype‐specific risk factors among HR‐negative BC. Our previous studies also suggested more commonalities than specificities among risk factors for traditionally four subtypes of BC.^21^ However, subtype‐specific risk factors would be the key points to determining the different subtypes. As observed in this study, low BMI, postmenopausal status, and history of abortion were independently associated with HER2‐zero BC, while OC and HRT were independently associated with HER2‐low BC. These results might suggest that HER2‐low BC would be more associated with exogenous hormones than HER2‐zero BC among HR‐negative BC. Moreover, previous studies suggested that HER2‐zero BC had a significantly higher prevalence of mutations in BRCA1/2 or other BC predisposition genes than HER2‐low BC.^9^, ^12^, ^40^, ^41^, ^51^ In addition to the above pathogenic genetic mutations, this study also supported the different subtype‐specific molecular landscapes of BC based on genetic susceptibility. Particularly, based on the distribution and the interaction between ERS and PRS, HER2‐low BC seemed to be less susceptible to risk factors and genetic susceptibility than HER2‐zero BC. Further studies are needed to validate these results in the future, and further novel markers are needed to better distinguish HER2‐low BC from HER2‐zero BC.

In addition to the above findings, some limitations also deserved attention in this study. First, as mentioned in the method, due to lack of ISH testing to reclassify BC with IHC 2+ as true HER2‐low BC or HER2‐positive BC, the current results would inevitably incur misclassification of HER2‐low BC and should be explained with caution.^52^, ^53^, ^54^, ^55^ Second, only selected SNPs were genotyped and would bias the whole picture of the subtype‐specific molecular landscape of BC. Although more and more SNPs have been identified to be significantly associated with BC based on GWAS, most SNPs were originally from European or American ancestors. All selected SNPs were identified and validated in a large sample size of Chinese or Asian females. Therefore, the current results would provide valuable significance for BC control in China and other East Asian females with similar genetic backgrounds. Thirdly, no further data are available to validate the current results, especially for the subtype‐specific risk factors and genetic susceptibility. Further studies with more sophisticated designs are needed not only to validate the current results.

## 5 CONCLUSIONS

In conclusion, due to a larger proportion, less clinical heterogeneity, better prognosis, and less susceptibility to risk factors, HER2‐low BC should be suggested as a potentially independent subtype of BC from traditionally HER2‐negative BC and should deserve more attention than HER2‐zero BC, especially in HR‐negative BC. Moreover, further studies are needed not only to improve the definition of HER2‐low BC, but also to precisely identify HER2‐low BC who will potentially benefit from novel ADC agents.

## AUTHOR CONTRIBUTIONS

**Lu Zheng:** Conceptualization (lead); data curation (lead); formal analysis (lead); writing – original draft (lead); writing – review and editing (equal). **Yunmeng Zhang:** Data curation (supporting); writing – original draft (supporting); writing – review and editing (equal). **Zhipeng Wang:** Data curation (supporting). **Huan Wang:** Conceptualization (supporting). **Chunfang Hao:** Writing – review and editing (supporting). **Chenyang Li:** Writing – review and editing (supporting). **Yanrui Zhao:** Data curation (supporting). **Zhangyan Lyu:** Writing – review and editing (supporting). **Fangfang Song:** Data curation (supporting). **Kexin Chen:** Conceptualization (supporting); resources (equal). **Yubei Huang:** Conceptualization (supporting); data curation (supporting); resources (equal); writing – review and editing (supporting). **Fengju Song:** Resources (equal).

## FUNDING INFORMATION

This work was supported by the Chinese National Key Research and Development Project (No. 2021YFC2500400), National Natural Science Foundation of China (81974439), and Tianjin Health Committee Foundation (TJWJ2021MS008). Tianjin Science and Technology Committee Foundation (18JCQNJC80300).

## CONFLICT OF INTEREST STATEMENT

The authors declare that they have no competing interests.

## ETHICS APPROVAL AND CONSENT TO PARTICIPATE

The Tianjin Medical University Cancer Institute and Hospital research ethics board approved the study (Ek2018002), and the written informed consent was acquired from each participant or their guardian.

## CONSENT FOR PUBLICATION

All authors have approved the manuscript and agree with its submission.

## Supporting information


Tables S1–S9.

## Data Availability

Individual‐level data is not freely available and future collaboration are welcome. For cooperation application, please contact Prof. Fengju Song [songfengju@163.com].
