# Supplementary material for: Comparisons of clinical characteristics, prognosis, epidemiological factors, and genetic susceptibility between HER2‐low and HER2‐zero breast cancer among Chinese females
Source: Cancer Med. 2023 Jun 30;12(14):14937–48. doi: 10.1002/cam4.6129 (PMC10417066; doi:10.1002/cam4.6129)
Supplement: Supplementary file 1 — Tables S1–S9. [file CAM4-12-14937-s001.docx]

**Supplementary table 1. Summary of 23 GWAS-identified SNPs reported in the original study.**

| SNPs | Chromosome | Gene | Allele | Position (bp) | OR (95%CI) | PMID |
| --- | --- | --- | --- | --- | --- | --- |
| SNPs initially reported in Asians | | |  |  |  |  |
| rs2046210 | 6q25.1 | *ESR1* | G/A | 151990059 | 1.29 (1.21-1.37) | 19219042 |
| rs4784227 | 16q12.1 | *TOX3* | T/C | 51156689 | 1.25 (1.20-1.31) | 20585626 |
| rs10822013 | 10q21.2 | *ZNF365* | C/T | 63921983 | 1.10 (1.06-1.15) | 21908515 |
| rs7107217 | 11q24.3 | *BARX2* | C/A | 128978900 | 1.08 (1.05-1.11) | 22383897 |
| rs9485372 | 6q25.1 | *TAB2* | A/G | 149650567 | 0.90 (0.87-0.92) | 22383897 |
| rs10474352 | 5q14.3 | *ARRDC3* | C/T | 90767981 | 1.09 (1.06-1.12) | 25038754 |
| rs2290203 | 15q26.1 | *PRC1* | G/A | 89313071 | 1.08 (1.05-1.11) | 25038754 |
| rs4951011 | 1q32.1 | *ZC3H11A* | G/A | 202032954 | 1.09 (1.06-1.12) | 25038754 |
| SNPs initially reported in Europeans | | |  |  |  |  |
| rs1219648 | 10q26.13 | *FGFR2* | G/A | 123336180 | 1.31 (1.25-1.37) | 17529973 |
| rs10941679 | 5p12 | *MRPS30* | A/G | 44742255 | 1.19 (1.13-1.26) | 18438407 |
| rs4973768 | 3p24.1 | *SLC4A7* | C/T | 27391017 | 1.11 (1.08-1.13) | 19330027 |
| rs10771399 | 12p11 | *PTHLH* | A/G | 28046347 | 0.85 (0.83-0.88) | 22267197 |
| rs1292011 | 12q24 | *MED13L* | A/G | 114320905 | 0.92 (0.91-1.03) | 22267197 |
| rs616488 | 1p36 | *PEX14* | A/G | 10488802 | 0.94 (0.90-0.98) | 23535729 |
| rs4849887 | 2q14.2 | *INHBB* | C/T | 120961592 | 0.90 (0.84-0.96) | 23535729 |
| rs16857609 | 2q35 | *DIRC3* | C/T | 218004753 | 1.09 (1.05-1.14) | 23535729 |
| rs6828523 | 4q34 | *ADAM29* | C/A | 176083001 | 0.89 (0.83-0.94) | 23535729 |
| rs1432679 | 5q33 | *EBF1* | T/C | 158176661 | 1.06 (1.02-1.10) | 23535729 |
| rs9693444 | 8p21 | *RPL17P33* | C/A | 29565535 | 1.07 (1.03-1.12) | 23535729 |
| rs6472903 | 8q21 | *HNF4G* | T/G | 76392856 | 0.88 (0.84-0.93) | 23535729 |
| rs17356907 | 12q22 | *NTN4* | A/G | 94551890 | 0.89 (0.85-0.93) | 23535729 |
| rs2236007 | 14q13 | *PAX9* | G/A | 36202520 | 0.88 (0.83-0.93) | 23535729 |
| rs17817449 | 16q12 | *FTO* | T/G | 52370868 | 0.95 (0.91-0.99) | 23535729 |

**Supplementary table 2. Multivariable Cox regression analysis for overall survival of HER2 zero and HER2 low-positive** **breast cancer (BC) by HR status.**

| Subgroups | Patients,  N | Deaths,  N(%) | Follow-up,  1000 PYs | Mortality rates,  per 1000 PYs | P value^*^ | P value^†^ |
| --- | --- | --- | --- | --- | --- | --- |
| Overall BC |  |  |  |  | **0.013** | 0.693 |
| HER2 zero | 4265 | 289(6.8) | 30.45 | 9.49 |  |  |
| HER2 low | 7646 | 444(5.8) | 38.00 | 11.68 |  |  |
| HR+ BC |  |  |  |  | **0.010** | 0.086 |
| HER2 zero | 3767 | 235(6.2) | 2.82 | 19.15 |  |  |
| HER2 low | 6135 | 328(5.3) | 7.29 | 15.91 |  |  |
| HR- BC |  |  |  |  | 0.222 | **0.031** |
| HER2 zero | 498 | 54(10.8) | 27.64 | 8.50 |  |  |
| HER2 low | 1511 | 116(7.7) | 30.71 | 10.68 |  |  |

Note: PY, person-year; HR (95%CI), hazard ratio (95% confidential interval); -, negative; +, positive. *, P value for log-rank test. †, adjusted age, pTNM, grade, Ki-67, chemotherapy, endocrinotherapy, radiotherapy; for overall BC, HR status was further adjusted.

**Supplementary table 3.** **Multivariable Cox regression analysis for overall survival of HER2 zero and HER2 low-positive** **breast cancer (BC) by HR status.**

| Characteristics* | | Overall BC(N=11,911) | | |  | HR+ BC(N=9,902) | | |  | HR- BC(N=2,009) | | |
| --- | --- | --- | --- | --- | --- | --- | --- | --- | --- | --- | --- | --- |
|  |  | N (%) | HR(95%CI) | P value |  | N (%) | HR(95%CI) | P value |  | N (%) | HR(95%CI) | P value |
| Age, years | ≤40 | 1,631 (13.7) | 1.04(0.81-1.32) | 0.781 |  | 72 (12.8) | 1.02(0.76-1.35) | 0.913 |  | 253 (12.6) | 1.06(0.65-1.73) | 0.808 |
|  | 41-50 | 4,016 (33.7) | Reference |  |  | 124 (22.1) | Reference |  |  | 582 (29.0) | Reference |  |
|  | 51-60 | 3,750 (31.5) | 0.79(0.64-0.97) | **0.027** |  | 144 (25.7) | 0.76(0.59-0.96) | **0.022** |  | 767 (38.2) | 0.916(0.60-1.40) | 0.685 |
|  | >60 | 2,500 (21.0) | 2.19(1.81-2.64) | **<0.001** |  | 221 (39.4) | 2.26(1.82-2.81) | **<0.001** |  | 405 (20.2) | 1.95(1.32-2.86) | **0.001** |
| HR | Negative | 2,009 (16.9) | Reference |  |  | - | - |  |  | - | - |  |
|  | Positive | 9,902 (83.1) | 0.67(0.55-0.81) | **<0.001** |  | - | - | **-** |  | - | - | - |
| pTNM | 0-I | 2,962(24.9) | Reference |  |  | 56 (13.6) | Reference |  |  | 490 (33.1) | Reference |  |
|  | II | 4,131 (34.7) | 1.91(1.44-2.53) | **<0.001** |  | 132 (32.0) | 1.66(1.21-2.27) | **0.002** |  | 711 (48.0) | 3.30(1.72-6.33) | **<0.001** |
|  | III-IV | 1,720 (14.4) | 6.62(5.00-8.77) | **<0.001** |  | 225 (54.5) | 6.31(4.63-8.60) | **<0.001** |  | 280 (18.9) | 8.53(4.33-16.79) | **<0.001** |
| Grade | G1/G2 | 6,006 (50.4) | Reference |  |  | 234 (87.3) | Reference |  |  | 765 (53.9) | Reference |  |
|  | G3 | 1,227 (10.3) | 1.12(0.87-1.46) | 0.382 |  | 34 (12.7) | 1.18(0.82-1.70) | 0.362 |  | 653 (46.1) | 0.96(0.65-1.41) | 0.819 |
| Ki-67 | ≤14% | 2,776 (23.3) | Reference |  |  | 129 (22.9) | Reference |  |  | 131 (6.5) | Reference |  |
|  | >14% | 9,135 (76.7) | 1.35(1.12-1.63) | **0.002** |  | 434 (77.1) | 1.40(1.14-1.71) | **0.001** |  | 1,878 (93.5) | 0.89(0.53-1.47) | 1.469 |
| Endocrinotherapy | No/Unknown | 7,456 (62.6) | Reference |  |  | 339 (67.4) | Reference |  |  | 1,655 (98.2) | Reference |  |
|  | Yes | 3,224 (27.1) | 0.90(0.74-1.08) | 0.251 |  | 164 (32.6) | 0.89(0.73-1.07) | 0.216 |  | 31 (1.8) | 1.13(0.36-3.58) | 0.837 |
| Chemotherapy | No/Unknown | 1,568 (13.2) | Reference |  |  | 114 (10.4) | Reference |  |  | 203 (10.3) | Reference |  |
|  | Yes | 10,131 (85.1) | 0.66(0.54-0.81) | **<0.001** |  | 444 (79.6) | 0.66(0.53-0.83) | **<0.001** |  | 1,768 (89.7) | 0.62(0.40-0.96) | **0.033** |
| Radiotherapy | No/Unknown | 8,194 (68.8) | Reference |  |  | 333 (62.5) | Reference |  |  | 1,318 (72.1) | Reference |  |
|  | Yes | 2,870 (24.1) | 1.23(1.02-1.47) | **0.029** |  | 200 (37.5) | 1.22(0.99-1.50) | 0.059 |  | 510 (27.9) | 1.25(0.84-1.84) | 0.269 |
| HER2 | HER2 zero | 4,265 (35.8) | Reference |  |  | 235 (41.7) | Reference |  |  | 498 (24.8) | Reference |  |
|  | HER2 low | 7,646 (64.2) | 1.03(0.88-1.21) | 0.693 |  | 328 (58.3) | 1.17(0.98-1.40) | 0.086 |  | 1,511 (75.2) | 0.69(0.50-0.97) | **0.031** |

Note:*, missing values in the index variable were not shown.

**Supplementary table 4.** **Distribution of epidemiological factors between HER2-zero breast cancer (BC), HER2-low BC, and healthy controls.**

| Risk Factors^*^ | | Control  (N=5,653) | Overall BC | | | |  | HR+ BC | | | |  | HR- BC | | | |
| --- | --- | --- | --- | --- | --- | --- | --- | --- | --- | --- | --- | --- | --- | --- | --- | --- |
|  |  |  | HER2-zero  (N=4,265) | P  value | HER2-low  (N=7,646) | P  value |  | HER2-zero  (N=3,767) | P  value | HER2-low  (N=6,135) | P  value |  | HER2-zero  (N=498) | P  value | HER2-low  (N=1,511) | P  value |
| BMI, kg/m^2^ | <18.5 | 79(1.4) | 65(1.5) | **<0.001** | 121(1.6) | **<0.001** |  | 54(1.4) | **<0.001** | 95(1.5) | **<0.001** |  | 11(2.2) | **<0.001** | 26(1.7) | **<0.001** |
|  | 18.5-23.9 | 2,413(42.7) | 1,416(33.2) |  | 2,690(35.2) |  |  | 1,243(33.0) |  | 2,142(34.9) |  |  | 173(34.7) |  | 548(36.3) |  |
|  | 24.0-27.9 | 2,411(42.6) | 1,311(30.7) |  | 2,568(33.6) |  |  | 1,137(30.2) |  | 2,046(33.3) |  |  | 174(34.9) |  | 522(34.5) |  |
|  | ≥28.0 | 750(13.3) | 690(16.2) |  | 1,196(15.6) |  |  | 612(16.2) |  | 979(16.0) |  |  | 78(15.7) |  | 217(14.4) |  |
| Smoking | No | 5,448(96.4) | 3,707(86.9) | **<0.001** | 6,728(88.0) | **<0.001** |  | 3,253(86.4) | **<0.001** | 5,411(88.2) | **<0.001** |  | 454(91.2) | **<0.001** | 1,317(87.2) | **<0.001** |
|  | Yes | 205(3.6) | 403(9.4) |  | 634(8.3) |  |  | 369(9.8) |  | 493(8.0) |  |  | 34(6.8) |  | 141(9.3) |  |
| History of  BBD | No | 4,375(77.4) | 2,677(62.8) | **<0.001** | 4,135(54.1) | **<0.001** |  | 2,394(63.6) | **<0.001** | 3,312(54.0) | **<0.001** |  | 283(56.8) | **<0.001** | 823(54.5) | **<0.001** |
|  | Yes | 1,278(22.6) | 1,375(32.2) |  | 2,892(37.8) |  |  | 1,194(31.7) |  | 2,325(37.9) |  |  | 181(36.3) |  | 567(37.5) |  |
| Family history  of BC | No | 5,509(97.5) | 4,024(94.3) | **<0.001** | 7,215(94.4) | **<0.001** |  | 3,552(94.3) | **<0.001** | 5,802(94.6) | **<0.001** |  | 472(94.8) | **<0.001** | 1,413(93.5) | **<0.001** |
|  | Yes | 144(2.5) | 241(5.7) |  | 431(5.6) |  |  | 215(5.7) |  | 333(5.4) |  |  | 26(5.2) |  | 98(6.5) |  |
| Age at  menarche | >14 years | 3,209(56.8) | 2,063(48.4) | **<0.001** | 3,603(47.1) | **<0.001** |  | 1,825(48.4) | **<0.001** | 2,881(47.0) | **<0.001** |  | 238(47.8) | **<0.001** | 722(47.8) | **<0.001** |
|  | ≤14 years | 2,444(43.2) | 2,076(48.7) |  | 3,491(45.7) |  |  | 1,843(48.9) |  | 2,813(45.9) |  |  | 233(46.8) |  | 678(44.9) |  |
| Menopause  status | Premenopausal | 1,941(34.3) | 1,999(46.9) | **<0.001** | 3,340(43.7) | **<0.001** |  | 1,788(47.5) | **<0.001** | 2,814(45.9) | **<0.001** |  | 211(42.4) | **<0.001** | 526(34.8) | **<0.001** |
|  | Postmenopausal | 3,712(65.7) | 2,190(51.3) |  | 4,105(53.7) |  |  | 1,923(51.0) |  | 3,165(51.6) |  |  | 267(53.6) |  | 940(62.2) |  |
| Abortion | Never | 1,882(33.3) | 1,120(26.3) | **<0.001** | 2,035(26.6) | **<0.001** |  | 998(26.5) | **<0.001** | 1,615(26.3) | **<0.001** |  | 122(24.5) | **<0.001** | 420(27.8) | **<0.001** |
|  | Ever | 3,771(66.7) | 2,974(69.7) |  | 5,188(67.9) |  |  | 2,625(69.7) |  | 4,175(68.1) |  |  | 349(70.1) |  | 1,013(67.0) |  |
| Oral  contraception | Never | 5,115(90.5) | 3,306(77.5) | **<0.001** | 5,654(73.9) | **<0.001** |  | 2,921(77.5) | **<0.001** | 4,540(74.0) | **<0.001** |  | 385(77.3) | **<0.001** | 1,114(73.7) | **<0.001** |
|  | Ever | 538(9.5) | 544(12.8) |  | 918(12.0) |  |  | 486(12.9) |  | 739(12.0) |  |  | 58(11.6) |  | 179(11.8) |  |
| HRT | Never | 5,491(97.1) | 3,665(85.9) | **<0.001** | 6,288(82.8) | **<0.001** |  | 3,235(85.9) | **<0.001** | 5,059(82.5) | **<0.001** |  | 430(86.3) | 0.165 | 1,229(81.3)) | **<0.001** |
|  | Ever | 162(2.9) | 1,96(4.6) |  | 303(4.0) |  |  | 178(4.7) |  | 233(3.8) |  |  | 18(3.6) |  | 70(4.6) |  |

Note:*, missing values in the index variable were not shown. BBD, benign breast disease; HRT, hormone replacement treatment.

**Supplementary table 5. Adjusted relative risks of HER2-zero and HER2-low breast cancer (BC) by HR status with epidemiological factors.**

| Risk Factors* | | Overall BC | | | |  | HR+ BC | | | |  | HR- BC | | | |
| --- | --- | --- | --- | --- | --- | --- | --- | --- | --- | --- | --- | --- | --- | --- | --- |
|  |  | HER2 zero | | HER2 low | |  | HER2 zero | | HER2 low | |  | HER2 zero | | HER2 low | |
|  |  | OR(95%CI) | P Value | OR(95%CI) | P Value |  | OR(95%CI) | P Value | OR(95%CI) | P Value |  | OR(95%CI) | P Value | OR(95%CI) | P Value |
| BMI, kg/m^2^ | <18.5 | 1.36(0.94-1.97) | 0.106 | 1.37(1.00-1.89) | 0.052 |  | 1.20(0.81-1.78) | 0.370 | 1.33(0.95-1.87) | 0.100 |  | 2.43(1.22-4.87) | **0.012** | 1.49(0.91-2.47) | 0.117 |
|  | 18.5-23.9 | Ref. |  | Ref. |  |  | Ref. |  | Ref. |  |  | Ref. |  | Ref. |  |
|  | 24.0-27.9 | 1.03(0.93-1.14) | 0.621 | 1.03(0.95-1.12) | 0.498 |  | 1.01(0.91-1.13) | 0.840 | 1.04(0.95-1.14) | 0.371 |  | 1.18(0.92-1.50) | 0.190 | 1.00(0.86-1.16) | 0.946 |
|  | ≥28.0 | 1.79(1.56-2.04) | **<0.001** | 1.57(1.39-1.76) | **<0.001** |  | 1.78(1.54-2.04) | **<0.001** | 1.64(1.45-1.86) | **<0.001** |  | 1.82(1.33-2.47) | **<0.001** | 1.30(1.06-1.60) | **0.011** |
| Smoking | No | Ref. |  | Ref. |  |  | Ref. |  | Ref. |  |  | Ref. |  | Ref. |  |
|  | Yes | 3.25(2.69-3.93) | **<0.001** | 2.85(2.39-3.39) | **<0.001** |  | 3.39(2.79-4.11) | **<0.001** | 2.75(2.29-3.30) | **<0.001** |  | 1.97(1.28-3.04) | **0.002** | 2.97(2.30-3.84) | **<0.001** |
| History of BBD | No | Ref. |  | Ref. |  |  | Ref. |  | Ref. |  |  | Ref. |  | Ref. |  |
|  | Yes | 1.75(1.58-1.94) | **<0.001** | 2.48(2.28-2.71) | **<0.001** |  | 1.69(1.52-1.89) | **<0.001** | 2.46(2.25-2.70) | **<0.001** |  | 2.14(1.70-2.68) | **<0.001** | 2.46(2.13-2.84) | **<0.001** |
| Family history of BC | No | Ref. |  | Ref. |  |  | Ref. |  | Ref. |  |  | Ref. |  | Ref. |  |
|  | Yes | 2.23(1.76-2.83) | **<0.001** | 2.34(1.90-2.88) | **<0.001** |  | 2.24(1.76-2.87) | **<0.001** | 2.30(1.85-2.86) | **<0.001** |  | 2.07(1.27-3.38) | **0.003** | 2.58(1.91-3.48) | **<0.001** |
| Age at menarche, years | >14 | Ref. |  | Ref. |  |  | Ref. |  | Ref. |  |  | Ref. |  | Ref. |  |
|  | ≤14 | 1.16(1.05-1.27) | **0.003** | 1.07(0.99-1.16) | 0.092 |  | 1.17(1.071.30) | **0.001** | 1.06(0.98-1.16) | 0.152 |  | 1.01(0.82-1.26) | 0.898 | 1.08(0.94-1.24) | 0.268 |
| Menopause status | Premenopausal | Ref. |  | Ref. |  |  | Ref. |  | Ref. |  |  | Ref. |  | Ref. |  |
|  | Postmenopausal | 0.56(0.51-0.62) | **<0.001** | 0.63(0.58-0.69) | **<0.001** |  | 0.54(0.49-0.59) | **<0.001** | 0.58(0.53-0.63) | **<0.001** |  | 0.77(0.61-0.96) | **0.019** | 0.94(0.82-1.09) | 0.427 |
| Abortion | Never | Ref. |  | Ref. |  |  | Ref. |  | Ref. |  |  | Ref. |  | Ref. |  |
|  | Ever | 1.21(1.10-1.35) | **<0.001** | 1.12(1.03-1.22) | **0.011** |  | 1.19(1.07-1.33) | **0.002** | 1.13(1.03-1.24) | **0.008** |  | 1.35(1.05-1.75) | **0.019** | 1.01(0.87-1.18) | 0.907 |
| Oral contraception | Never | Ref. |  | Ref. |  |  | Ref. |  | Ref. |  |  | Ref. |  | Ref. |  |
|  | Ever | 1.55(1.34-1.78) | **<0.001** | 1.51(1.34-1.72) | **<0.001** |  | 1.57(1.35-1.82) | **<0.001** | 1.52(1.33-1.74) | **<0.001** |  | 1.33(0.96-1.85) | 0.082 | 1.40(1.14-1.72) | **0.002** |
| HRT | Never | Ref. |  | Ref. |  |  | Ref. |  | Ref. |  |  | Ref. |  | Ref. |  |
|  | Ever | 1.59(1.25-2.01) | **<0.001** | 1.33(1.08-1.65) | **0.009** |  | 1.63(1.27-2.08) | **<0.001** | 1.27(1.01-1.59) | **0.038** |  | 1.29(0.77-2.19) | 0.335 | 1.46(1.06-2.03) | **0.022** |

Note:*, missing values in the index variable were not shown.

**Supplementary table 6. Unadjusted relative risks of HER2-zero and HER2-low breast cancer (BC) by HR status with GWAS-identified SNPs.**

| SNPs | Overall BC | | | |  | HR+ BC | | | |  | HR- BC | | | |
| --- | --- | --- | --- | --- | --- | --- | --- | --- | --- | --- | --- | --- | --- | --- |
|  | HRE2 zero | | HER2 low | |  | HRE2 zero | | HER2 low | |  | HRE2 zero | | HER2 low | |
|  | OR(95%CI) | P value | OR(95%CI) | P value |  | OR(95%CI) | P value | OR(95%CI) | P value |  | OR(95%CI) | P value | OR(95%CI) | P value |
| rs2046210 | 1.20(1.12-1. 30) | **<0.001** | 1.24(1.15-1.33) | **<0.001** |  | 1.17(1.09-1.27) | **<0.001** | 1.19(1.10-1.28) | **<0.001** |  | 1.48(1.21-1.80) | **<0.001** | 1.43(1.25-1.63) | **<0.001** |
| rs4784227 | 1.27(1.17-1.38) | **<0.001** | 1.22 (1.13-1.32) | **<0.001** |  | 1.29(1.19-1.41) | **<0.001** | 1.27(1.17-1.38) | **<0.001** |  | 1.09(0.87-1.37) | 0.443 | 1.03(0.88-1.20) | 0.754 |
| rs1219648 | 1.25(1.16-1.34) | **<0.001** | 1.24(1.16-1.33) | **<0.001** |  | 1.26(1.17-1.36) | **<0.001** | 1.32(1.22-1.42) | **<0.001** |  | 1.12(0.91-1.37) | 0.282 | 0.98(0.85-1.12) | 0.736 |
| rs4973768 | 1.15(1.06-1.25) | **0.002** | 1.14(1.05-1.24) | **0.002** |  | 1.17(1.07-1.28) | **0.001** | 1.13(1.04-1.24) | **0.006** |  | 0.98(0.77-1.26) | 0.894 | 1.16(0.99-1.36) | 0.075 |
| rs17356907 | 1.18(1.08-1.29) | **<0.001** | 1.12(1.03-1.22) | **0.007** |  | 1.16(1.06-1.27) | **0.001** | 1.12(1.02-1.22) | **0.019** |  | 1.39(1.07-1.80) | **0.013** | 1.15(0.97-1.35) | 0.107 |
| rs10771399 | 1.14(1.04-1.25) | **0.006** | 1.15(1.05-1.25) | **0.002** |  | 1.13(1.03-1.24) | **0.013** | 1.14(1.04-1.26) | **0.006** |  | 1.24(0.95-1.61) | 0.111 | 1.16(0.97-1.38) | 0.100 |
| rs10474352 | 1.14(1.06-1.22) | **<0.001** | 1.14(1.06-1.22) | **<0.001** |  | 1.16(1.07-1.25) | **<0.001** | 1.17(1.08-1.26) | **<0.001** |  | 0.97(0.80-1.18) | 0.771 | 1.02(0.90-1.17) | 0.728 |
| rs2290203 | 1.10(1.02-1.18) | **0.011** | 1.09(1.02-1.17) | **0.015** |  | 1.11(1.03-1.20 | **0.006** | 1.07(1.00-1.16) | 0.064 |  | 0.98(0.80-1.20) | 0.869 | 1.15(1.01-1.32) | **0.039** |
| rs4951011 | 1.10(1.02-1.20) | **0.014** | 1.11(1.03-1.19) | **0.009** |  | 1.10(1.02-1.20) | **0.020** | 1.13(1.04-1.22) | **0.005** |  | 1.12(0.90-1.39) | 0.327 | 1.03(0.89-1.19) | 0.707 |
| rs10941679 | 1.16(1.07-1.24) | **<0.001** | 1.09(1.02-1.17) | **0.016** |  | 1.18(1.10-1.28) | **<0.001** | 1.11(1.03-1.20) | **0.008** |  | 0.92(0.75-1.13) | 0.431 | 1.02(0.89-1.17) | 0.762 |
| rs4849887 | 1.04(0.95-1.15) | 0.409 | 1.16(1.06-1.28) | **0.002** |  | 1.04(0.94-1.15) | 0.492 | 1.19(1.07-1.32) | **0.001** |  | 1.10(0.84-1.44) | 0.512 | 1.07(0.89-1.28) | 0.495 |
| rs7107217 | 1.08(1.00-1.16) | 0.054 | 1.09(1.01-1.17) | **0.019** |  | 1.08(1.00-1.16) | 0.066 | 1.09(1.01-1.18) | **0.034** |  | 1.08(0.88-1.33) | 0.454 | 1.10 (0.96-1.26) | 0.190 |
| rs1292011 | 1.10(1.00-1.20) | **0.040** | 1.07(0.99-1.17) | 0.101 |  | 1.09(0.99-1.20) | 0.071 | 1.08 (0.99-1.19) | 0.092 |  | 1.18(0.92-1.53) | 0.196 | 1.04(0.88-1.23) | 0.655 |
| rs2236007 | 1.15(1.06-1.25) | **0.001** | 1.07(0.99-1.16) | 0.076 |  | 1.17(1.07-1.28) | **<0.001** | 1.09(1.00-1.19) | **0.044** |  | 0.97(0.78-1.22) | 0.815 | 1.01(0.87-1.17) | 0.951 |
| rs9693444 | 1.11(1.02-1.20) | **0.014** | 1.06(0.98-1.14) | 0.151 |  | 1.11(1.03-1.21) | **0.011** | 1.06(0.97-1.15) | 0.186 |  | 1.04(0.83-1.30) | 0.744 | 1.06(0.91-1.23) | 0.466 |
| rs10822013 | 1.06(0.98-1.14) | 0.142 | 1.08(1.01-1.16) | **0.033** |  | 1.06(0.98-1.14) | 0.157 | 1.09(1.01-1.18) | **0.020** |  | 1.06(0.86-1.29) | 0.606 | 1.02(0.89-1.17) | 0.769 |
| rs9485372 | 1.07(0.99-1.15) | 0.071 | 1.08(1.01-1.16) | **0.033** |  | 1.07(0.99-1.15) | 0.098 | 1.09(1.01-1.17) | **0.030** |  | 1.10(0.90-1.35) | 0.354 | 1.05(0.91-1.20) | 0.528 |
| rs1432679 | 1.04(0.96-1.12) | 0.350 | 1.08(1.00-1.16) | **0.038** |  | 1.04(0.96-1.12) | 0.393 | 1.03(0.96-1.12) | 0.396 |  | 1.05(0.85-1.30) | 0.630 | 1.29(1.11-1.49) | **0.001** |
| rs616488 | 1.06(0.98-1.15) | 0.160 | 1.04(0.96-1.12) | 0.312 |  | 1.05(0.97-1.14) | 0.233 | 1.03(0.95-1.12) | 0.446 |  | 1.13(0.90-1.41) | 0.299 | 1.07(0.92-1.24) | 0.380 |
| rs7697216 | 1.08(0.99-1.17) | 0.077 | 1.02(0.94-1.10) | 0.661 |  | 1.07(0.98-1.17) | 0.110 | 1.02(0.93-1.11) | 0.712 |  | 1.12(0.89-1.42) | 0.330 | 1.02(0.88-1.20) | 0.763 |
| rs16857609 | 1.07(0.99-1.15) | 0.072 | 1.03(0.96-1.10) | 0.409 |  | 1.07(0.99-1.15) | 0.093 | 1.02(0.95-1.10) | 0.570 |  | 1.09(0.89-1.34) | 0.416 | 1.06(0.93-1.22) | 0.399 |

**Supplementary table 7. Distribution of ERS for HER2 zero and HER2 low-positive breast cancer (BC) by HR status compared to healthy controls.**

| ERS(%) | Control | Overall BC | |  | HR+ BC | |  | HR- BC | |
| --- | --- | --- | --- | --- | --- | --- | --- | --- | --- |
|  |  | HER2 zero | HER2 low |  | HER2 zero | HER2 low |  | HER2 zero | HER2 low |
| 20 | 2,924(51.7) | 904(30.4) | 1,598(30.8) |  | 794(30.4) | 1,260(30.2) |  | 102(28.6) | 335(32.8) |
| 40 | 1,835(32.5) | 1,088(36.6) | 2,209(42.5) |  | 952(36.4) | 1,772(42.4) |  | 139(38.9) | 420(41.2) |
| 60 | 765(13.5) | 792(26.7) | 1,158(22.3) |  | 701(26.8) | 960(23.0) |  | 87(24.4) | 215(21.1) |
| 80 | 115(2.0) | 160(5.4) | 210(4.0) |  | 143(5.5) | 169(4.0) |  | 25(7.0) | 43(4.2) |
| 100 | 14(0.3) | 28(0.9) | 22(0.4) |  | 25(0.9) | 16(0.4) |  | 4(1.1) | 7(0.7) |

**Supplementary table 8. Distribution of PRS for HER2 zero and HER2 low-positive breast cancer (BC) by HR status compared to healthy controls.**

| PRS(%) | Control | Overall BC | |  | HR+ BC | |  | HR- BC | |
| --- | --- | --- | --- | --- | --- | --- | --- | --- | --- |
|  |  | HER2 zero | HER2 low |  | HER2 zero | HER2 low |  | HER2 zero | HER2 low |
| 20 | 28(0.5) | 61(3.1) | 152(6.8) |  | 51(2.9) | 121(6.7) |  | 10(5.1) | 31(6.8) |
| 40 | 1,083(19.1) | 523(26.5) | 820(36.5) |  | 475(26.7) | 643(35.9) |  | 48(24.4) | 106(23.3) |
| 60 | 2,765(48.9) | 991(50.2) | 866(38.5) |  | 889(50.0) | 673(37.5) |  | 81(41.1) | 213(46.7) |
| 80 | 1,716(30.4) | 370(18.7) | 377(16.7) |  | 336(18.9) | 330(18.4) |  | 48(24.4) | 79(17.3) |
| 100 | 61(1.1) | 29(1.5) | 34(1.5) |  | 26(1.5) | 26(1.5) |  | 10(5.0) | 27(5.9) |

**Supplementary table 9. Interaction of ERS and PRS on the risks of HER2-zero and HER2-low breast cancer (BC) by HR status.**

| Quartiles  of PRS | Quartiles  of ERS | HER2-zero BC | | | | | | | |  | HER2-low BC | | | | | | | |
| --- | --- | --- | --- | --- | --- | --- | --- | --- | --- | --- | --- | --- | --- | --- | --- | --- | --- | --- |
|  |  | Overall BC | |  | HR+ BC | |  | HR- BC | |  | Overall BC | |  | HR+ BC | |  | HR- BC | |
|  |  | OR  (95%CI) | P  value |  | OR  (95%CI) | P  value |  | OR  (95%CI) | P  value |  | OR  (95%CI) | P  value |  | OR  (95%CI) | P  value |  | OR  (95%CI) | P  value |
| Q1 | Q1 | Ref. |  |  | Ref. |  |  | Ref. |  |  | Ref. |  |  | Ref. |  |  | Ref. |  |
|  | Q2 | 1.83(1.42-2.36) | <0.001 |  | 1.85(1.41-2.42) | <0.001 |  | 1.71(0.86-3.38) | 0.123 |  | 1.49(1.16-1.90) | 0.002 |  | 1.50(1.14-1.97) | 0.003 |  | 1.48(0.87-2.51) | 0.147 |
|  | Q3 | 3.24(2.49-4.22) | <0.001 |  | 3.48(2.65-4.58) | <0.001 |  | 1.75(0.84-3.67) | 0.137 |  | 2.77(2.15-3.56) | <0.001 |  | 2.62(1.99-3.45) | <0.001 |  | 3.18(1.88-5.37) | <0.001 |
|  | Q4 | 3.63(2.76-4.79) | <0.001 |  | 3.76(2.81-5.04) | <0.001 |  | 1.83(0.81-4.11) | 0.144 |  | 3.50(2.69-4.55) | <0.001 |  | 3.63(2.71-4.85) | <0.001 |  | 3.64(2.15-6.15) | <0.001 |
| Q2 | Q1 | 1.43(1.1-1.87) | 0.008 |  | 1.47(1.11-1.95) | 0.007 |  | 1.09(0.52-2.27) | 0.827 |  | 1.38(1.08-1.77) | 0.011 |  | 1.32(1.00-1.74) | 0.049 |  | 1.76(1.05-2.94) | 0.032 |
|  | Q2 | 2.45(1.86-3.22) | <0.001 |  | 2.59(1.94-3.45) | <0.001 |  | 1.47(0.63-3.40) | 0.372 |  | 2.47(1.91-3.19) | <0.001 |  | 2.47(1.87-3.27) | <0.001 |  | 2.35(1.38-4.02) | 0.002 |
|  | Q3 | 3.37(2.48-4.58) | <0.001 |  | 3.32(2.40-4.58) | <0.001 |  | 3.07(1.48-6.34) | 0.002 |  | 4.15(3.16-5.45) | <0.001 |  | 3.94(2.93-5.31) | <0.001 |  | 5.12(3.00-8.75) | <0.001 |
|  | Q4 | 5.66(4.22-7.58) | <0.001 |  | 5.92(4.35-8.04) | <0.001 |  | 4.00(1.89-8.43) | <0.001 |  | 5.48(4.14-7.25) | <0.001 |  | 5.65(4.17-7.65) | <0.001 |  | 5.16(2.97-8.96) | <0.001 |
| Q3 | Q1 | 1.93(1.47-2.53) | <0.001 |  | 2.03(1.53-2.69) | <0.001 |  | 1.13(0.51-2.54) | 0.76 |  | 1.79(1.39-2.32) | <0.001 |  | 1.66(1.25-2.21) | 0.001 |  | 2.71(1.49-4.98) | 0.001 |
|  | Q2 | 3.40(2.57-4.50) | <0.001 |  | 3.69(2.76-4.94) | <0.001 |  | 1.45(0.57-3.69) | 0.436 |  | 2.64(2.00-3.48) | <0.001 |  | 2.49(1.84-3.39) | <0.001 |  | 3.92(2.15-7.14) | <0.001 |
|  | Q3 | 5.96(4.41-8.05) | <0.001 |  | 6.26(4.59-8.53) | <0.001 |  | 3.06(1.36-6.92) | 0.007 |  | 4.94(3.69-6.61) | <0.001 |  | 4.29(3.13-5.88) | <0.001 |  | 6.00(3.15-11.42) | <0.001 |
|  | Q4 | 6.28(4.61-8.55) | <0.001 |  | 6.76(4.90-9.34) | <0.001 |  | 3.31(1.41-7.74) | 0.006 |  | 5.83(4.32-7.87) | <0.001 |  | 6.42(4.62-8.93) | <0.001 |  | 5.32(2.56-11.05) | <0.001 |
| Q4 | Q1 | 2.42(1.81-3.24) | <0.001 |  | 2.63(1.95-3.55) | <0.001 |  | 0.94(0.35-2.55) | 0.902 |  | 1.86(1.39-2.49) | 0.001 |  | 2.05(1.51-2.79) | <0.001 |  | 1.29(0.72-2.32) | 0.396 |
|  | Q2 | 4.28(3.18-5.77) | <0.001 |  | 4.52(3.32-6.17) | <0.001 |  | 2.92(1.25-6.81) | 0.013 |  | 3.21(2.38-4.33) | <0.001 |  | 3.42(2.49-4.71) | <0.001 |  | 2.69(1.56-4.66) | <0.001 |
|  | Q3 | 9.02(6.43-12.66) | <0.001 |  | 9.27(6.53-13.15) | <0.001 |  | 4.74(2.08-10.79) | <0.001 |  | 7.10(5.07-9.93) | <0.001 |  | 7.51(5.29-10.67) | <0.001 |  | 5.86(3.27-10.48) | <0.001 |
|  | Q4 | 10.20(7.29-14.28) | <0.001 |  | 10.71(7.55-15.17) | <0.001 |  | 7.00(3.14-15.63) | <0.001 |  | 7.84(5.60-10.99) | <0.001 |  | 8.84(6.19-12.62) | <0.001 |  | 5.70(3.26-9.98) | <0.001 |
